# Could Water and Sanitation Shortfalls Exacerbate SARS-CoV-2 Transmission Risks?

**DOI:** 10.4269/ajtmh.20-0462

**Published:** 2020-06-09

**Authors:** Erkison E. Odih, Ayorinde O. Afolayan, IfeOluwa Akintayo, Iruka N. Okeke

**Affiliations:** 1 Department of Pharmaceutical Microbiology, Faculty of Pharmacy, University of Ibadan, Ibadan, Nigeria;; 2 Department of Veterinary and Animal Sciences, Faculty of Health and Medical Sciences, University of Copenhagen, Copenhagen, Denmark

## Abstract

SARS-CoV-2, the etiologic agent of COVID-19, is shed in stool. SARS coronaviruses have been detected in wastewater during outbreaks in China, Europe, and the United States. In this perspective, we outline the risk fecal shedding poses at locations without safely managed sanitation, as in most of Nigeria where we work. We believe that feco-oral transmission could occur if community transmission becomes high and sustained in densely populated cities without proper sanitation in Nigeria and many other African and Asian settings. In the absence of basic sanitation, or where existing sanitation is not safely managed, groundwater, which is often drawn up from wells and boreholes for drinking and household use, can become contaminated with enteric bacteria and viruses from fecal matter. Endemic and epidemic transmission of multiple feco-oral pathogens via this route continues to be documented in areas without safely managed sanitation, and, therefore, the risk of SARS-CoV-2 transmission needs to be evaluated, tracked, and forestalled in such settings. We suggest that fecal matter from treatment facilities and recovered patients should be carefully and properly disposed. Furthermore, environmental surveillance of SARS-CoV-2 in wastewater and accumulated human waste, as well as efforts to mitigate the virus’ entry into unprotected household water sources, should be a priority part of the COVID-19 response in settings without safely managed sanitation for the duration of the pandemic.

Every effort must be deployed to limit continued spread of the etiologic agent of the COVID-19 pandemic. Mounting evidence shows that SARS-CoV-2 is amplified in the gastrointestinal tracts of infected people, excreted in stool, and detectable in wastewater at high levels^1–3^ (Rimoldi et al., 2020; medRxiv preprint doi: https://doi.org/10.1101/2020.05.01.20086009). We are concerned that, in areas without safely managed sanitation, drinking and household water supplies could become contaminated with the virus. This potential risk of feco-oral transmission is highest in densely populated urban centers.

SARS-CoV-2 has largely been transmitted via respiratory droplets and fomites from infected persons to the respiratory systems of susceptible individuals.^4^ However, the virus replicates in gut enterocytes^1^ and is detected in stool from patients with severe or mild COVID-19, as well as from presymptomatic and asymptomatic individuals.^2,3^ Recovered COVID-19 patients may continue to shed virus for as long as 42 days after symptoms have ceased, even after they test negative by conventional respiratory tests.^3,5,6^

Considerable concern has been expressed in the literature that the feco-oral transmission potential for SARS-CoV-2 places endoscopists, caregivers of diapered children who shed the virus,^7^ and fecal transplant recipients^8^ at high risk of contracting the infection. For intestinal SARS-CoV-2 to transmit via fecal matter, it would have to be viable when shed and persist in the environment until a count greater than an oral infective dose is ingested by a susceptible individual. The cycle could potentially be shortcut by direct dissemination of fecal matter inadvertently from person-to-person or by pests like flies and cockroaches, or it could be broken if wastewater or domestic water treatment inactivates SARS-CoV-2. Because wastewater treatment eradicates SARS-CoV-2 and most of the worst affected countries have robust water purification systems, community feco-oral transmission has been less extensively discussed.

Feco-orally transmitted pathogens are endemic in Nigeria, which is among the top five countries worldwide contributing to diarrhea-derived under-five mortality.^9^ A principal reason why the burden from feco-oral pathogens is so high is that for most Nigerians, sewage systems are nonfunctional, incompletely functional, or nonexistent. Of six major northern Nigeria cities conducting polio virus environmental surveillance, only Abuja operates a sewage plant.^10^ In Lagos, there are multiple sewage treatment plants, but their performance is suboptimal, and they therefore pose a risk of enteric pathogen transmission to surrounding areas.^11,12^ Sewage handling capacity has not grown in tandem with the explosive and continued growth of this megacity so that coverage does not extend to all residents. This situation prevails in many African urban centers and in some south Asian settings: between 60% and 95% of African city dwellers are not connected to sewerage and instead use a range of autonomous solutions or resort to open defecation.^13^ As a result, fecal matter can be deposited into the open environment, pour from toilets unconnected to sewerage into surface water, or be buried underground in soakaways and pits from where, if these receptacles are not adequately protected, it can seep into shallow wells used for irrigation, drinking, and household purposes.^10,13–15^

According to the WHO/UNICEF, 663 million people in Africa and Asia do not have access to safe water, and diarrheal disease is a major cause of illness and death in those populations.^16^ In recent years, surveillance of household water at a number of African and Asian locations has revealed frequently found indicators of recent fecal contamination, such as *Escherichia coli*, or outright pathogens, including enteric viruses. In each case, links have been made to human or animal open defecation, proximal latrines, or improperly processed wastewater.^17–19^ In those settings, even pathogens known not to persist or thrive in the environment are among those recovered from contaminated household water or causing outbreaks.^20,21^ Although there are as yet no reports of transmission of SARS-CoV-2 via sewage or fecal matter in settings without safely managed sanitation, or recovery from household water, these examples demonstrate that feco-oral transmission by endemic pathogenic organisms is commonplace in these settings.

There is now convincing evidence, from countries with adequate sanitation, that SARS-CoV-2 is present in feces, around toilets, and in wastewater.^2,22,23^ Both SARS-CoV and SARS-CoV-2 nucleic acid have been detected in wastewater during outbreaks.^22,24,25^ Replicable virus is less commonly reported, although it is less commonly sought, and has been found. In laboratory studies, Wang et al.^25^ found that SARS-CoV, the agent of the 2003 SARS epidemic, remains viable in water for 14 days at 4°C but for only 2 days at 20°C, suggesting that survival in tropical regions may be inadequate to sustain viability in stored water. However, coronaviruses are protected by organic matter,^26^ and this will greatly affect their survival under real-world conditions. SARS-CoV-2 RNA has been detected in substantial concentrations in wastewater and downstream water bodies.^27^ Rimoldi et al. reported that SARS-CoV-2 is susceptible to wastewater treatment and that viral infectiveness in wastewater is negligible; viral RNA was amplified in untreated but not treated wastewater.^28^ Zhang and coworkers^29^ in another preprint, however, found the China CDC-recommended sodium hypochlorite treatment of wastewater to be ineffective for the removal of SARS-CoV-2 RNA. These studies await peer review, and further investigation is needed to clarify risks.

As noted by Lodder and de Roda Husman,^22^ early finding of a COVID-19 case in the United States with no known exposure to an infected case suggests that a form other than human-to-human respiratory transmission of COVID-19 may be possible. Additional evidence comes from a recently published systematic review from Wuhan, which spotlighted a small number of patients with diarrhea but no respiratory symptoms.^30^ However, most patients in this pandemic who could have had the opportunity to be infected feco-orally to date have also been exposed to respiratory droplets or fomites; thus, the magnitude of the risk is challenging to gauge. Feco-oral transmission nonetheless remains a valid, if untested, hypothesis.^1,2,22,26,30–33^ Our review of the evidence suggests that the risk of this mode of transmission in communities without basic sanitation may be high. Unfortunately, countries without effective sanitation and water purification are also those least likely to have the wherewithal to detect live virus in environmental samples (detection of viral nucleic acid does not infer infectivity) and therefore measure this risk.^10,34^

As at the time of writing, most African and Asian cities without basic or safely managed sanitation had reported relatively few COVID-19 cases. However, case numbers are increasing, and, as they rise, the viral load in untreated fecal waste pools could escalate. This is particularly true of urban settings experiencing rapid rises in case numbers such as Lagos and Kano, two Nigerian megacities. As at May 23, 2020, the Nigerian Centre for Disease Control had confirmed 3,357 and 883 cases in these states, respectively, together representing 56.3% of the number of cases nationally. Because of occupancy pressures on isolation facilities, most Nigerian cities have erected makeshift isolation and treatment facilities for patients who have tested positive, to supplement the few facilities that were available at the start of the pandemic. These new facilities often lie outside hospitals that manage their own wastewater. The same pressures on treatment facilities mean that SARS-CoV-2–infected persons in Nigeria are discharged as soon as two consecutive respiratory swabs test negative: symptom free but likely shedding the virus when they return to their communities. Other factors could additionally combine to alter the risk of feco-oral SARS-CoV-2 transmission. In Kathmandu, Nepal, where *Salmonella enterica* Typhi and Paratyphi have been shown to leach into the municipal water system, breaches occur more heavily during the rains.^35^ We note that African countries on the upswing of their COVID-19 epidemics are just beginning the rainy season. Rainy season sewage overflows can overwhelm even properly managed wastewater plants, leading to heightened enteric virus transmission.^36^ On the other hand, it is possible that only very high counts of SARS-CoV-2 would yield orally infectious doses. Thus, feco-oral transmission may only occur when the epidemic reaches an as yet unknown threshold. Either way, those at risk within those settings are poor urban communities and informal settlements, which have the worst sanitation options and access to health care. Individuals could become infected even if they were able to implement physical distancing recommendations, which themselves are a challenge, and need to be decisively protected from fecal SARS-CoV-2.^37^

Possible options for halting feco-oral SARS-CoV-2 transmission are disinfection of known open defecation sites, intensifying handwashing messages, encouraging boiling or chemical treatment of household water, and explicitly treating waste from isolation and treatment facilities. Safer sewage management should be instituted or reinstituted, as priority where possible. These include ensuring that standard operating procedures are followed, and there are no interruptions in sewage decontamination, as well as quality assurance to ensure that decontamination goals are met.^11^ Stalled or slowed sanitation projects should be expedited, and new ones could be explored. Individuals who have to work in or close to wastewater handling facilities, particularly those operating suboptimally, should be informed of their risk and provided with protection where feasible. In those situations, as research from the 2003 SARS outbreak demonstrated, aerosolized virus poses a risk for respiratory transmission in addition to any feco-oral risk that may exist.^38,39^

On the positive side, fecal shedding of SARS-CoV-2 can be exploited for community surveillance of wastewater or human waste using similar methods that would be required for risk evaluation.^22,40^ Enteric pathogen, polio, and antimicrobial resistance environmental surveillance could be leveraged, where these have been initiated,^10,41,42^ but sites with no access to sewerage, typically not used for surveillance, must also be included. In high-risk settings, waste and wastewater-based epidemiology could help balance sampling biases inherent in case- and contact-tracing–based human testing for COVID-19 and consequently predict prevalence.^42,43^ It would also preemptively identify epidemic foci and ascertain the exact risk of community transmission via the fecal–oral route. Indeed, it could represent a dedicated strategy to protect the poor and marginalized in whom outbreaks in this pandemic have typically been detected with significant lags.^44^ Although much is focused on the current emergency, the potential risk from feco-oral SARS-CoV transmission should motivate and even initiate concrete steps toward lasting wastewater and sewage systems wherever possible. This would leave a post–COVID-19 development legacy that could impact disease transmission, extend the value of other disease control strategies,^45^ and improve the quality of life in the long term.

## References

[1] Lamers MM 2020 SARS-CoV-2 productively infects human gut enterocytes. Science 10.1126/science.abc1669. PMC7199907 32358202

[2] Chen Y 2020 The presence of SARS-CoV-2 RNA in the feces of COVID-19 patients. J Med Virol 10.1002/jmv.25825. 32243607

[3] Xu Y 2020 Characteristics of pediatric SARS-CoV-2 infection and potential evidence for persistent fecal viral shedding. Nat Med 26: 502–505. 32284613 10.1038/s41591-020-0817-4 PMC7095102

[4] WHO, 2020 Modes of Transmission of Virus Causing COVID-19: Implications for IPC Precaution Recommendations: Scientific Brief, 27 March 2020. Geneva, Switzerland: World Health Organization.

[5] Wu Y 2020 Prolonged presence of SARS-CoV-2 viral RNA in faecal samples. Lancet Gastroenterol Hepatol 5: 434–435. 32199469 10.1016/S2468-1253(20)30083-2 PMC7158584

[6] Jiang X Luo M Zou Z Wang X Chen C Qiu J, 2020 Asymptomatic SARS-CoV-2 infected case with viral detection positive in stool but negative in nasopharyngeal samples lasts for 42 days. J Med Virol 10.1002/jmv.25941. PMC7264508 32330309

[7] Bonato G Dioscoridi L Mutignani M, 2020 Faecal-oral transmission of SARS-COV-2: practical implications. Gastroenterology 10.1053/j.gastro.2020.03.066. PMC7194731 32247692

[8] Green CA Quraishi MN Shabir S Sharma N Hansen R Gaya DR Hart AL Loman NJ Iqbal TH, 2020 Screening faecal microbiota transplant donors for SARS-CoV-2 by molecular testing of stool is the safest way forward. Lancet Gastroenterol Hepatol 5: 531. 32240618 10.1016/S2468-1253(20)30089-3 PMC7225406

[9] Troeger C Blacker BF Khalil IA Rao PC Cao S Zimsen SR Albertson SB Stanaway JD Deshpande A Abebe Z, 2018 Estimates of the global, regional, and national morbidity, mortality, and aetiologies of diarrhoea in 195 countries: a systematic analysis for the global burden of disease study 2016. Lancet Infect Dis 18: 1211–1228. 30243583 10.1016/S1473-3099(18)30362-1 PMC6202444

[10] Johnson Muluh T Hamisu AW Craig K Mkanda P Andrew E Adeniji J Akande A Musa A Ayodeji I Nicksy G, 2016 Contribution of environmental surveillance toward interruption of poliovirus transmission in Nigeria, 2012–2015. J Infect Dis 213: S131–S135. 26908747 10.1093/infdis/jiv767 PMC4818559

[11] Longe E Ogundipe A, 2010 Assessment of wastewater discharge impact from a sewage treatment plant on lagoon water, Lagos, Nigeria. Res J Appl Sci Eng Technol 2: 274–282.

[12] Adeniji JA Faleye TO, 2014 Isolation and identification of enteroviruses from sewage and sewage-contaminated water in Lagos, Nigeria. Food Environ Virol 6: 75–86. 24566762 10.1007/s12560-014-9137-5

[13] Manzo OL Saidou H Illiassou SA Idrissa ST, 2015 Assessment of domestic wastewater management practices in the communal district I of Maradi city, Niger Republic. J Geosci Environ Prot 3: 57–65.

[14] Doron A Jeffrey R, 2018 Waste of a Nation: Garbage and Growth in India. Cambridge, MA: Harvard University Press.

[15] Editorial, 2018 We need to talk about crapping. Nat Microbiol 3: 1189. 30356153 10.1038/s41564-018-0287-3

[16] WHO, 2015 Progress on Sanitation and Drinking Water: 2015 Update and MDG Assessment. Geneva, Switzerland: World Health Organization: WHO/UNICEF Joint Water Supply Sanitation Monitoring Programme.

[17] Moreira NA Bondelind M, 2017 Safe drinking water and waterborne outbreaks. J Water Health 15: 83–96. 28151442 10.2166/wh.2016.103

[18] Gwimbi P George M Ramphalile M, 2019 Bacterial contamination of drinking water sources in rural villages of Mohale Basin, Lesotho: exposures through neighbourhood sanitation and hygiene practices. Environ Health Prev Med 24: 33. 31092211 10.1186/s12199-019-0790-z PMC6521341

[19] Karkey A 2010 The burden and characteristics of enteric fever at a healthcare facility in a densely populated area of Kathmandu. PLoS One 5: e13988. 21085575 10.1371/journal.pone.0013988 PMC2981554

[20] Gupta BP Haselbeck A Kim JH Marks F Saluja T, 2018 The dengue virus in Nepal: gaps in diagnosis and surveillance. Ann Clin Microbiol Antimicrob 17: 32. 30008269 10.1186/s12941-018-0284-7 PMC6047123

[21] Kabwama SN 2017 A large and persistent outbreak of typhoid fever caused by consuming contaminated water and street-vended beverages: Kampala, Uganda, January–June 2015. BMC Public Health 17: 23. 28056940 10.1186/s12889-016-4002-0 PMC5216563

[22] Lodder W de Roda Husman AM, 2020 SARS-CoV-2 in wastewater: potential health risk, but also data source. Lancet Gastroenterol Hepatol 5: 533–534. 32246939 10.1016/S2468-1253(20)30087-X PMC7225404

[23] Liu Y 2020 Aerodynamic analysis of SARS-CoV-2 in two Wuhan hospitals. Nature 10.1038/s41586-020-2271-3. 32340022

[24] La Rosa G Bonadonna L Lucentini L Kenmoe S Suffredini E, 2020 Coronavirus in water environments: occurrence, persistence and concentration methods - a scoping review. Water Res 179: 115899. 32361598 10.1016/j.watres.2020.115899 PMC7187830

[25] Wang XW 2005 Concentration and detection of SARS coronavirus in sewage from Xiao Tang Shan hospital and the 309th hospital. J Virol Methods 128: 156–161. 15964082 10.1016/j.jviromet.2005.03.022 PMC7112879

[26] Gundy PM Gerba CP Pepper IL, 2009 Survival of coronaviruses in water and wastewater. Food Environ Virol 1: 10.

[27] Mallapaty S, 2020 How sewage could reveal true scale of coronavirus outbreak. Nature 580: 176–177. 10.1038/d41586-020-00973-x 32246117

[28] Rimoldi SG 2020 10.1101/2020.05.01.20086009.

[29] Zhang D 2020 10.1101/2020.04.28.20083832.

[30] Tian Y Rong L Nian W He Y, 2020 Review article: gastrointestinal features in COVID-19 and the possibility of faecal transmission. Aliment Pharmacol Ther 51: 843–851. 32222988 10.1111/apt.15731 PMC7161803

[31] Heller L Mota CR Greco DB, 2020 COVID-19 faecal-oral transmission: are we asking the right questions? Sci Total Environ 729: 138919. 32353720 10.1016/j.scitotenv.2020.138919 PMC7182518

[32] Yeo C Kaushal S Yeo D, 2020 Enteric involvement of coronaviruses: is faecal-oral transmission of SARS-CoV-2 possible? Lancet Gastroenterol Hepatol 5: 335–337. 32087098 10.1016/S2468-1253(20)30048-0 PMC7130008

[33] Meng X Huang X Zhou P Li C Wu A, 2020 Alert for SARS-CoV-2 infection caused by fecal aerosols in rural areas in China. Infect Control Hosp Epidemiol 10.1017/ice.2020.114. PMC7174849 32252855

[34] Okeke IN, 2011 Divining without Seeds: The Case for Strengthening Laboratory Medicine in Africa. Ithaca, NY: ILR/ Cornell University Press.

[35] Karkey A 2016 The ecological dynamics of fecal contamination and *Salmonella Typhi* and *Salmonella Paratyphi* A in municipal Kathmandu drinking water. PLoS Negl Trop Dis 10: e0004346. 26735696 10.1371/journal.pntd.0004346 PMC4703202

[36] Rodríguez RA Gundy PM Rijal GK Gerba CP, 2012 The impact of combined sewage overflows on the viral contamination of receiving waters. Food Environ Virol 4: 34–40. 23412766 10.1007/s12560-011-9076-3

[37] Corburn J 2020 Slum health: arresting COVID-19 and improving well-being in urban informal settlements. J Urban Health 10.1007/s11524-020-00438-6. PMC7182092 32333243

[38] Yu IT Li Y Wong TW Tam W Chan AT Lee JH Leung DY Ho T, 2004 Evidence of airborne transmission of the severe acute respiratory syndrome virus. N Engl J Med 350: 1731–1739. 15102999 10.1056/NEJMoa032867

[39] McKinney KR Gong YY Lewis TG, 2006 Environmental transmission of SARS at amoy gardens. J Environ Health 68: 26–30. 16696450

[40] Senghore M Savi MK Gnangnon B Hanage WP Okeke IN, 2020 Leveraging Africa’s preparedness towards the next phase of the COVID-19 pandemic. Lancet Glob Health 10.1016/S2214-109X(20)30234-5. PMC7255144 32416768

[41] Hendriksen RS 2019 Global monitoring of antimicrobial resistance based on metagenomics analyses of urban sewage. Nat Commun 10: 1124. 30850636 10.1038/s41467-019-08853-3 PMC6408512

[42] Hendriksen RS 2019 Pathogen surveillance in the informal settlement, Kibera, Kenya, using a metagenomics approach. PLoS One 14: e0222531. 31600207 10.1371/journal.pone.0222531 PMC6786639

[43] Murakami M Hata A Honda R Watanabe T, 2020 Letter to the editor: wastewater-based epidemiology can overcome representativeness and stigma issues related to COVID-19. Environ Sci Technology 54: 5311. 10.1021/acs.est.0c02172 32323978

[44] von Braun J Zamagni S Sorondo MS, 2020 The moment to see the poor. Science 368: 214. 32299923 10.1126/science.abc2255

[45] Delahoy MJ Cárcamo C Ordoñez L Vasquez V Lopman B Clasen T Gonzales GF Steenland K Levy K, 2020 Impact of rotavirus vaccination varies by level of access to piped water and sewerage: an analysis of childhood clinic visits for diarrhea in Peru, 2005–2015. Pediatr Infect Dis J 10.1097/INF.0000000000002702. PMC7368830 32332220

